# Prevalence of reproductive tract infections and the predictive value of girls’ symptom-based reporting: findings from a cross-sectional survey in rural western Kenya

**DOI:** 10.1136/sextrans-2015-052371

**Published:** 2016-01-27

**Authors:** Emily Kerubo, Kayla F Laserson, Newton Otecko, Collins Odhiambo, Linda Mason, Elizabeth Nyothach, Kelvin O Oruko, Ashley Bauman, John Vulule, Clement Zeh, Penelope A Phillips-Howard

**Affiliations:** 1 Center for Global Health Research, Kenya Medical Research Institute (KEMRI) Kisumu, Kisumu, Kenya; 2 Division of Global Health Protection, Center for Global Health, Centers for Disease Control and Prevention (CDC), Atlanta, USA; 3 Department of Clinical Sciences, Liverpool School of Tropical Medicine, Liverpool, UK; 4 Division of HIV/AIDS Prevention, Centers for Disease Control and Prevention, Kisumu, Kenya

**Keywords:** ADOLESCENT, AFRICA, DIAGNOSIS, REPRODUCTIVE HEALTH, SEXUAL HEALTH

## Abstract

**Objectives:**

Reproductive tract infections (RTIs), including sexually acquired, among adolescent girls is a public health concern, but few studies have measured prevalence in low-middle-income countries. The objective of this study was to examine prevalence in rural schoolgirls in Kenya against their reported symptoms.

**Methods:**

In 2013, a survey was conducted in 542 adolescent schoolgirls aged 14–17 years who were enrolled in a menstrual feasibility study. Vaginal self-swabbing was conducted after girls were interviewed face-to-face by trained nurses on symptoms. The prevalence of girls with symptoms and laboratory-confirmed infections, and the sensitivity, specificity, positive and negative predictive values of symptoms compared with laboratory results, were calculated.

**Results:**

Of 515 girls agreeing to self-swab, 510 answered symptom questions. A quarter (24%) reported one or more symptoms; most commonly vaginal discharge (11%), pain (9%) or itching (4%). Laboratory tests confirmed 28% of girls had one or more RTI. Prevalence rose with age; among girls aged 16–17 years, 33% had infections. Bacterial vaginosis was the most common (18%), followed by *Candida albicans* (9%), *Chlamydia trachomatis* (3%), *Trichomonas vaginalis* (3%) and *Neisseria gonorrhoeae* (1%). Reported symptoms had a low sensitivity and positive predictive value. Three-quarters of girls with bacterial vaginosis and *C. albicans*, and 50% with *T. vaginalis* were asymptomatic.

**Conclusions:**

There is a high prevalence of adolescent schoolgirls with RTI in rural Kenya. Public efforts are required to identify and treat infections among girls to reduce longer-term sequelae but poor reliability of symptom reporting minimises utility of symptom-based diagnosis in this population.

**Trial registration number:**

ISRCTN17486946.

## Introduction

An estimated half a billion new cases of curable sexually transmitted infections occur each year globally,^1^ ^2^ with women being more susceptible and bearing the greatest burden of these infections. Reproductive tract infections (RTIs), including those sexually acquired, have historically been labelled a ‘silent’ epidemic among females, contributing to gynaecological morbidity and maternal mortality globally, including low-middle-income countries (LMICs).^1^ Untreated infections can lead to pelvic inflammatory diseases, ectopic pregnancy, infertility, cervical cancer, foetal loss or infant health complications, with a threefold to fivefold increase in risk of HIV acquisition and transmission.^1^ ^w1^ Bacterial vaginosis is associated with increased risk for acquisition of HIV,^3^ ^w2^ promotes viral shedding among HIV-infected females^4^ and increases susceptibility for other infections such as gonorrhoea, trichomoniasis and herpes simplex virus type-2 (HSV-2).^5–8^ ^w3^ Adolescent females are vulnerable to bacterial vaginosis infection,^1^ ^w4^ with an association between human papillomavirus infection, bacterial vaginosis and cervicitis.^9^ This is compounded by adolescent girls’ heightened susceptibility to HIV, for biological as well as socio-cultural reasons,^10^ in western Kenya, for example, young females have a sixfold greater risk of contracting HIV compared with young males.^11^

Although research on the burden of RTI among adolescent females in LMIC is limited, WHO estimates 80–90% of the global burden lies in LMICs.^1^ In Kenya, one in every five youths 15–24 years report sexual debut before the age of 15 years.^w5^ While HIV prevalence has fallen nationally, it remains static in rural parts of the former Nyanza Province^w5^ and rises among girls during adolescence.^11^ For the region, RTI prevalence is also high, with a third of urban adolescent girls aged 15–19 years diagnosed with *Trichomonas vaginalis,*^12^ and a smaller proportion with other infections (9% *Chlamydia trachomatis*; 2% *Neisseria gonorrhoeae*, 3% syphilis).^13^ However, data are sparse on the prevalence of RTI among post-pubescent girls in rural African settings.

Symptom assessment has commonly been used to prescribe treatment of RTI, and a simplified algorithm to guide health workers in the implementation of syndromic management of RTI has been developed.^14^ ^w6^ This was intended to define cause among the symptomatic rather than as a screening tool in the general population. Experts advocate supplementary point-of-care testing because of the poor predictive value of symptoms alone.^15^ Symptom guidelines at the national level have also been questioned in western Kenya when compared with laboratory diagnosis.^w7^ While menstrual studies among adolescent girls describe a high prevalence of RTI, seemingly due to poor hygiene,^w8^ most relied on reported symptoms.^16^ However, a study among women found symptom-based reporting was higher than laboratory-confirmed RTIs illustrating again the limitation of symptom reporting.^16^ The objective of this study was to measure the prevalence of RTI among adolescent schoolgirls in a menstrual feasibility study in rural western Kenya and to calculate the predictive value of girls’ symptom-based reporting against laboratory-confirmed infections.

## Methods

### Study setting

The population under study are resident in the Kenyan Medical Research Institute (KEMRI) and Centers for Disease Control and Prevention's health and demographic surveillance system (HDSS),^w9^ in Gem subcounty, Siaya County, western Kenya. This rural population are almost exclusively comprised of the Luo ethnic group who are mainly farmers.^w9^

### Study design

Cross-sectional, laboratory-supported, RTI survey nested within a menstrual feasibility study.

### Menstrual feasibility study

The ‘parent’ study, conducted August 2012 to November 2013, was a randomised controlled pilot examining the feasibility of menstrual solutions for girls in rural primary schools in western Kenya. In total, 30 of 62 schools participating in a baseline water, sanitation and hygiene survey reached eligibility for participation.^17^ No schools withdrew. All girls in study schools were enrolled if resident in the study area, were aged 14–16 years at enrolment, in classes 5–8, had no debilitation precluding participation and had experienced three or more menses. We estimated 669 participants followed to outcome provided 76% power to detect a 50% reduction in outcome (dropout), with an intra-cluster correlation coefficient of 0.01 and design effect of 1.21. Of 1005 target girls screened, 766 were enrolled and 40 (4%) refused, with the remaining 199 ineligible. Of the 766, 542 were in school for the cross-sectional RTI survey conducted in November 2013. Attrition (224 girls; 29.2%) prior to the RTI survey was due to withdrawal (2.1%), migration (12.4%), dropout (7.9%) and participants in class 8 exiting after their exams in October (6.8%).

### RTI cross-sectional survey methods

Study nurses, trained by a gynaecologist, privately questioned participants on a list of RTI-associated signs and symptoms before sample collection (see online supplementary form S1). All survey tools were pilot tested and nurses explained symptoms when interviewing. Symptoms covered urinary complaints, pain or bleed during sexual intercourse, vaginal discharge, malodorous or fishy smell, genital itching/irritation, lower abdominal or vaginal pain or both, and abnormal bleeding (heavy or between periods).^14^ ^w6^ ^w10^

Nurses trained participants to obtain their own vaginal swab.^18^ Samples were transported daily to the KEMRI laboratory, prepared and analysed for *Candida albicans*, bacterial vaginosis, *T. vaginalis*, *C. trachomatis* and *N. gonorrhoeae* using standard methods*.*^w11^ Bacterial vaginosis was defined as a Nugent Score of 7–10. Girls with a laboratory-confirmed diagnosis were treated by study nurses according to Kenyan national guidelines.

### Statistical analysis

Laboratory-confirmed infections were the measured outcome, and signs and symptoms the predictor variables, with age, socio-economic status (SES), age at menarche and prior use of sanitary pads also assessed as potential confounders. Data on SES were missing for 78 participants and were excluded from specific SES-related analysis. The prevalence of girls with individual infections and the 95% CI were calculated, then aggregated into all RTI. The prevalence of girls reporting symptoms were calculated, then aggregated into any symptom reported. Girls’ ages were collapsed into younger (<16 years) and older (>16 years) age groups. SES of participants was available from routine household surveys in the HDSS,^w9^ dichotomising quintiles into the poorest (1–2) and least poor (3–5). Differences due to age, SES, age of menarche and any prior sanitary pad use were assessed using Pearson's χ^2^ test. The sensitivity, specificity, positive predictive value (PPV) and negative predictive value (NPV) of symptoms alone, and then pooled, were computed for predicting each laboratory-confirmed (gold standard) infection, along with their 95% CI. Data analysis was performed using SPSS v.21.0.

## Results

Of 542 adolescent schoolgirls present in school and approached for this study, 515 (95%) participated in the vaginal swabbing; 25 declined saying they were menstruating (including at a second follow-up) and 2 refused. Age at survey ranged from 14 to 17 years, with a mean of 15.2 years (SD 0.6 year). Participants’ median SES index was 4 (range 1–5), with 17% falling into the poorest two quintiles. Girls’ mean age of menarche was 13.6 years (SD 0.9 year), with 83.5% reporting some prior use of sanitary pads. Less than 1% of girls reported they were currently sexually active (past month), while 46 (9%) answered the question on symptoms occurring during sex. Of 515 girls who self-swabbed for laboratory confirmation of RTI diagnoses, 510 (99%) answered questions on symptoms.

A quarter of participants reported one or more symptoms (table 1). Most common among these was vaginal discharge, followed by pain or itching. There was no difference in overall symptoms reported by age, SES, age at menarche or prior use of sanitary pads. However, younger (<16 years) girls were twofold less likely to report vaginal discharge than girls aged >16 years (p=0.002; table 1). Overlapping of symptoms was common, with two-thirds of girls reporting two or more symptoms in addition to itching or sore vagina, malodorous smell, burning urine or bleeding between menses (see online supplementary table S2).

**Table 1:** Prevalence of girls with reported symptoms by age group at survey

	Prevalence (95% CIs) of girls with reported symptoms							Age comparison
		14–15 years		16–17 years		Total	Ratios	χ^2^, p value
		370		140		510*		
Any symptom	82/370	22.2 (18.0 to 26.4)	40/140	28.6 (21.1 to 36.1)	122/510	23.9 (20.2 to 27.6)	0.78 (0.56 to 1.07)	2.29; 0.13
Heavy menstruation	25/370	6.8 (4.3 to 9.4)	8/140	5.7 (1.9 to 9.5)	33/510	6.5 (4.4 to 8.6)	1.18 (0.55 to 2.56)	0.18; 0.67
Between menses bleeding	15/370	4.1 (2.1 to 6.1)	5/140	3.6 (0.5 to 6.7)	20/510	3.9 (2.2 to 5.6)	1.14 (0.42 to 3.07)	0.06; 0.80
Burning urine	9/370	2.4 (0.8 to 4.0)	2/140	1.4 (−0.6 to 3.4)	11/510	2.2 (0.9 to 3.5)	1.70 (0.37 to 7.78)	0.49; 0.49
Frequency of urine	11/370	3.0 (1.3 to 4.7)	2/140	1.4 (−0.6 to 3.4)	13/510	2.5 (1.2 to 3.9)	2.08 (0.47 to 9.27)	0.98; 0.32
Abdominal/vaginal pain	30/370	8.1 (5.3 to 10.9)	15/140	10.7 (5.6 to 15.8)	45/510	8.8 (6.3 to 11.3)	0.76 (0.42 to 1.36)	0.86; 0.35
Pain during intercourse†	1/35	2.9 (−2.7 to 8.5)	1/11	9.1 (−7.9 to 26.1)	2/46	4.3 (−1.6 to 10.2)	0.31 (0.02to 4.62)	0.78; 0.38
Itchy/sore vagina‡	16/365	4.4 (2.3 to 6.5)	6/139	4.3 (0.9 to 7.7)	32/504	4.4 (2.6 to 6.2)	1.02 (0.41 to 2.54)	0.001; 0.94
Malodorous smell	5/364	1.4 (0.2 to 2.6)	4/139	2.9 (0.1 to 5.7)	9/503	1.8 (0.6 to 3.0)	0.48 (0.13 to 1.75)	1.3; 0.26
Vaginal discharge	29/367	7.9 (5.1 to 10.7)	34/139	17.3 (11.0 to 23.6)	53/506	10.5 (7.8 to 13.2)	0.46 (0.28 to 0.76)	9.4; 0.002

*Five participants reporting ‘don't know’ for all symptoms are excluded. †Sample limited to girls responding to symptoms of pain or bleeding during sexual intercourse (n=46). ‡4–6 girls did not answer questions on vaginal itching, smell and discharge.

Laboratory tests confirmed 28% of participants had at least one RTI, with bacterial vaginosis the most common diagnosis among 18% of girls (table 2). *T. vaginalis* was threefold higher in older compared with younger girls (p=0.03). A higher proportion of girls in the poorest SES quintiles were diagnosed with bacterial vaginosis or *C. albicans* (p=0.076).

**Table 2:** Prevalence of girls with laboratory-confirmed infections by age group

	Prevalence (95% CIs) of girls with laboratory diagnoses*							Age comparison
	14–15 years		16–17 years		Total		Ratios	χ^2^, p value
*Bacterial vaginosis*	61/374	16.3 (12.2 to 20.4)	33/141	1.12 (0.58 to 2.15)	94/515	18.3 (14.6 to 22.0)	0.70 (0.48 to 1.02)	3.45; 0.06
*Candida albicans*	33/373	8.8 (5.8 to 11.8)	11/139	0.75 (0.56 to 1.02)	44/512	8.6 (6.1 to 11.1)	1.12 (0.58 to 2.15)	0.11; 0.74
*Chlamydia trachomatis*	10/371	2.7 (1.1 to 4.4)	3/140	0.32 (0.11 to 0.93)	13/511	2.5 (1.2 to 3.9)	1.26 (0.35 to 4.50)	0.13; 0.73
*Trichomonas vaginalis*	6/373	1.6 (0.3 to 2.9)	7/139	0.76 (0.07 to 8.26)	13/512	2.5 (1.2 to 3.9)	0.32 (0.11 to 0.93)	4.81; 0.03
*Neisseria gonorrhoeae*	2/371	0.5 (−0.2 to 1.2)	1/140	0.55 (0.26 to 1.15)	3/511	0.6 (−0.1 to 1.3)	0.76 (0.07 to 8.26)	0.5; 0.82
All reproductive tract infections	98/374	26.2 (21.7 to 30.7)	47/141	0.70 (0.48 to 1.02)	145/515	28.2 (24.3 to 32.1)	0.79 (0.59 to 1.05)	2.57; 0.11

*2–4 tests spoiled.

Three-quarters of girls with bacterial vaginosis and *C. albicans* infections were asymptomatic, with slightly higher proportions of girls with *T. vaginalis, C. trachomatis* and *N. gonorrhoeae* reporting at least one symptom (table 3). In the analysis of sensitivity and PPV, between girls’ reporting symptoms and laboratory-confirmed bacterial vaginosis, malodorous smell achieved a PPV >50%, while vaginal itching was 32%. There was a low sensitivity and PPV for girls’ reporting symptoms associated with *C. trachomatis.* Pain or bleed during intercourse was predictive for *T. vaginalis* among the few infected girls acknowledging sexual intercourse. The low prevalence of girls with *N. gonorrhoeae* (<1%) limited analysis. Burning urine had a low PPV (table 3).

**Table 3:** Disease prediction of symptoms against laboratory diagnosis (95% CI) of reproductive tract infections

	Sensitivity		Specificity		PPV		NPV	
	n	% (95% CI)	n	% (95% CI)	N	% (95% CI)	n	% (95% CI)
Bacterial vaginosis								
Heavy menstruation	7/93	7.5 (3.1 to 14.9)	391/417	93.8 (91.0 to 95.9)	7/33	21.2 (9.0 to 38.9)	391/477	82.0 (78.2 to 85.3)
Between menses bleeding	4/93	4.3 (1.2 to 11.0)	401/417	96.2 (93.8 to 97.8)	4/20	20.0 (5.7 to 43.7)	401/490	81.8 (78.1 to 85.2)
Burning urine	2/93	2.2 (0.3 to 7.6)	408/417	97.8 (95.9 to 99.0)	2/11	18.2 (2.3 to 51.8)	408/499	81.8 (78.1 to 85.1)
Frequency of urine	4/93	4.2 (1.2 to 10.4)	408/417	97.8 (95.9 to 99.0)	4/13	30.8 (9.1 to 61.4)	408/497	82.1(78.2 to 85.3)
Abdominal/vaginal pain	10/93	10.7 (5.3 to 18.9)	382/417	91.6 (88.5 to 94.1)	10/45	22.2 (11.2 to 37.1)	382/465	82.2 (78.4 to 85.5)
Pain during intercourse*	1/6	16.7 (0.5 to 64.1)	39/40	97.5 (86.4 to 99.9)	1/2	50.0 (1.3 to 98.7)	39/44	88.6 (75.4 to 96.2)
Itchy/sore vagina†	7/92	7.6 (3.1 to 15.1)	397/412	96.4 (94.1 to 98.0)	7/22	31.8 (13.9 to 54.9)	397/482	82.4 (78.7 to 85.7)
Malodorous smell	5/91	5.5 (1.8 to 12.4)	408/412	99.0 (97.5 to 99.7)	5/9	55.6 (21.2 to 86.3)	408/494	82.6 (79.0 to 85.8)
Vaginal discharge	11/93	11.8 (6.1 to 20.2)	371/413	89.8 (86.5 to 92.6)	11/53	20.8 (10.8 to 34.1)	371/453	81.9 (78.0 to 85.3)
Any symptoms	26/93	28.0 (19.1 to 38.2)	321/417	77.0 (72.6 to 80.9)	26/122	21.3 (15.0 to 29.7)	321/388	82.7 (78.6 to 86.4)
*Candida albicans*								
Heavy menstruation	4/44	9.1 (2.5 to 21.7)	435/463	94.0 (91.4 to 95.9)	4/32	12.5 (3.5 to 29.0)	435/475	91.6 (88.7 to 93.9)
Between menses bleeding	1/44	2.2 (0.1 to 12.0)	444/563	95.9 (93.7 to 97.5)	1/20	5.0 (0.1 to24.9)	444/487	91.2 (88.3 to 93.5)
Burning urine	0/44	0.0 (0.0 to 8.4)	452/463	97.6 (95.8 to 98.8)	0/11	0.0 (0.0 to 28.5)	452/496	91.1 (88.3 to 93.5)
Frequency of urine	0/44	0.0 (0.0 to 8.0)	451/463	97.4 (95.5 to 98.7)	0/12	0.0 (0.0 to 26.5)	451/495	91.1 (88.3 to 93.5)
Abdominal/vaginal pain	6/44	13.6 (5.2 to 27.9)	424/463	91.6(88.7 to 93.9)	6/45	13.3 (5.5 to 26.8)	424/462	91.8 (88.9 to 94.1)
Pain during intercourse	0/8	0.0 (0.0 to 36.9)	36/38	94.7 (82.3 to 99.4)	0/2	0.0 (0.0 to 84.2)	36/44	81.8 (67.3 to 91.8)
Itchy/sore vagina	1/44	2.3 (0.1 to 12.0)	437/457	95.6 (93.3 to 97.3)	1/21	4.8 (0.1 to 23.8)	437/480	91.0 (88.1 to 93.4)
Malodorous smell	0/42	0.0 (0.0 to 8.4)	450/458	98.3 (96.6 to 99.2)	0/8	0.0 (0.0 to 36.9)	450/492	91.5 (88.6 to 93.8)
Vaginal discharge	3/43	7.0 (1.4 to 19.1)	410/460	89.1 (85.9 to 91.8)	3/53	5.7 (1.2 to 15.7)	410/450	91.1 (88.1 to 93.6)
Any symptoms	11/44	25.0 (13.2 to 40.3)	354/463	76.5 (72.3 to 80.3)	11/120	9.2 (4.7 to 15.8)	354/387	91.1 (88.2 to 94.1)
*Chlamydia trachomatis*								
Heavy menstruation	0/13	0.0 (0.0 to 24.7)	461/494	93.3 (90.6 to 95.4)	0/33	0.0 (0.0 to 10.6)	461/474	97.3 (95.4 to 98.5)
Between menses bleeding	0/13	0.0 (0.0 to 24.7)	475/494	96.2 (94.1 to 97.7)	0/19	0.0 (0.0 to 17.7)	475/488	97.3 (95.5 to 98.6)
Burning urine	1/13	7.7 (0.2 to 36.0)	484/494	98.0 (96.3 to 99.0)	1/11	9.1 (0.2 to 41.3)	484/496	97.6 (95.8 to 98.8)
Frequency of urine	0/13	0.0 (0.0 to 24.7)	481/494	97.4 (95.5 to 98.6)	0/13	0.0 (0.0 to 24.7)	481/494	97.4 (95.5 to 98.6)
Abdominal/vaginal pain	2/13	15.4 (1.9 to 45.5)	451/494	91.3 (88.5 to 93.6)	2/45	4.4 (0.5 to 15.2)	451/462	97.6 (95.8 to 98.8)
Pain during intercourse	0/1	0.0 (0.0 to 97.5)	43/45	95.6 (84.9 to 99.5)	0/2	0.0 (0.0 to 84.2)	43/44	97.7 (88.0 to 99.9)
Itchy/sore vagina	1/13	7.7 (0.2 to 36.0)	467/488	95.7 (93.5 to 97.3)	1/22	4.6 (0.1 to 22.8)	467/479	97.5 (95.7 to 98.7)
Malodorous smell	0/13	0.0 (0.0 to 24.7)	478/487	98.2 (96.5 to 99.2)	0/9	0.0 (0.0 to 33.6)	478/491	97.4 (95.5 to 98.6)
Vaginal discharge	2/13	15.4 (1.9 to 45.5)	439/490	89.6 (86.5 to 92.2)	2/53	3.8 (0.5 to 13.0)	439/450	97.6 (95.7 to 98.8)
Any symptoms	4/13	30.8 (9.1 to 61.4)	377/494	76.3 (72.3 to 80.0)	4/121	3.3 (0.9 to 8.3)	377/386	97.7 (95.6 to 98.9)
*Trichomonas vaginalis*								
Heavy menstruation	1/12	8.3 (0.2 to 38.5)	464/495	93.4 (90.8 to 95.4)	1/32	2.9 (0.1 to 15.3)	464/475	97.7 (95.9 to 98.8)
Between menses bleeding	0/12	0.0 (0.0 to 26.5)	475/495	96.0 (93.8 to 97.5)	0/20	0.0 (0.0 to 16.8)	475/487	97.5 (95.7 to 98.7)
Burning urine	1/12	8.3 (0.2 to 38.5)	485/495	98.0 (96.3 to 99.0)	1/11	9.1 (0.2 to 41.3)	485/496	97.8 (96.1 to 98.9)
Frequency of urine	0/12	0.0 (0.0 to 26.5)	483/495	97.6 (95.8 to 98.7)	0/12	0.0 (0.0 to 26.5)	483/495	97.6 (95.8 to 98.7)
Abdominal/vaginal pain	2/12	16.7 (2.1 to 48.4)	452/495	91.3 (88.5 to 93.6)	2/45	4.4 (0.5 to 15.2)	452/462	97.8 (96.1 to 99.0)
Pain during intercourse	2/2	100 (15.8 to 100)	44/44	100 (92.0 to 100)	2/2	100 (15.8 to 100)	44/44	100 (92.0 to 100)
Itchy/sore vagina	1/12	8.3 (0.2 to 38.5)	469/489	95.9 (93.8 to 97.5)	1/21	4.8 (0.1 to 23.8)	469/480	97.7 (95.9 to 98.9)
Malodorous smell	2/12	16.7 (2.1 to 48.4)	482/488	98.8 (97.3 to 100)	2/8	25.0 (3.2 to 65.1)	482/492	98.0 (96.3 to 100)
Vaginal discharge	2/12	16.7 (2.1 to 48.4)	440/491	89.6 (86.6 to 92.2)	2/53	3.8 (0.5 to 13.0)	440/450	97.8 (96.0 to 98.9)
Any symptoms	6/12	50.0 (21.1 to 78.9)	381/495	77.0 (73.0 to 80.6)	6/120	5.0 (1.9 to 10.6)	381/387	98.5 (96.7 to 99.4)
Neisseria gonorrhoeae*								
Heavy menstruation	1/3	33.3 (0.8 to 90.6)	472/504	93.7 (91.2 to 95.6)	1/33	3.0 (0.1 to 15.8)	472/474	99.6 (98.5 to 100)
Between menses bleeding	1/3	33.3 (0.8 to 90.6)	486/504	96.4 (94.4 to 97.9)	1/19	5.3 (0.1 to 26.0)	486/488	99.6 (98.5 to 100)
Burning urine	1/3	33.3 (0.8 to 90.6)	494/504	98.2 (96.4 to 99.0)	1/11	9.1 (0.2 to 41.3)	494/496	99.6 (98.6 to 100)
Frequency of urine	0/3	0.0 (0.0 to 70.6)	491/504	97.4 (95.6 to 98.6)	0/13	0.0 (0.0 to 24.7)	491/494	99.4 (98.6 to 99.9)
Abdominal/vaginal pain	1/3	33.3 (0.8 to 90.6)	460/504	91.3 (88.5 to 93.6)	1/45	2.2 (0.1 to 11.8)	460/462	99.6 (98.5 to 100)
Itchy/sore vagina	1/3	33.3 (0.8 to 90.6)	477/498	95.8 (93.6 to 97.4)	1/22	4.6 (0.1 to 22.8)	477/479	99.6 (98.5 to 100)
Malodorous smell	0/3	0.0 (0.0 to 70.7)	488/497	98.2 (96.6 to 99.1)	0/9	0.0 (0.0 to 33.6)	488/491	99.4 (98.2 to 100)
Vaginal discharge	1/3	33.3 (0.8 to 90.6)	448/500	89.6 (86.6 to 92.1)	1/53	1.9 (0.1 to 10.1)	448/450	99.6 (98.4 to 100)
Any symptoms	2/3	66.7 (9.4 to 99.1)	385/504	76.4 (72.3 to 80.0)	2/121	1.9 (0.2 to 5.8)	385/386	99.7 (98.6 to 100)

*Pain or bleed during intercourse was limited to 46 girls responding to symptoms of pain or bleeding during sexual intercourse (no data for *N. gonorrhoeae*).

†4–6 girls did not answer questions on vaginal itching, smell, discharge.

NPV, negative predictive value; PPV, positive predictive value.

## Discussion

This study demonstrates over a quarter of young adolescent schoolgirls in a rural African setting had a laboratory-diagnosed RTI. One-third of older aged (16–17 years) girls were infected. The largest contribution to the infection pool was from bacterial vaginosis. The predictive value of reported symptoms was generally very low. Despite this, there remains inadequate data on laboratory-confirmed cases of RTI among school-aged girls in Africa.

This study was nested in a cluster randomised controlled pilot study examining the feasibility of girls’ use of menstrual care products in schools. Sampling bias may have occurred as schools have been shown to be protective against sexual infections such as HIV,^19^ ^20^ resulting in a lower prevalence of RTI than among non-attending adolescent girls. We acknowledge use of some menstrual items may have potentially reduced some girls’ exposure to RTI,^16^ ^21^ with outcomes on the effect of different products currently under evaluation. While a high reliability of self-obtained swabs for RTI diagnosis has been demonstrated elsewhere,^18^ ^w12^ we note girls in our endline focus groups reported a minority of their peers avoided swabbing by spitting on the swabs. Such swabs were processed in the denominator and would have also possibly lowered the prevalence of girls with detected infections. Less than 1% of girls said they had sexual intercourse in the past month, and 9% answered a question on symptoms occurring during sex at face-to-face interview. This is an underestimate, as in a private self-completed survey 28% reported sexual exposure (unpublished). Studies in Kenya and Tanzania estimated up to a threefold higher prevalence of sexual exposure (including coerced) when girls self-completed surveys, for example, by computer.^22^ ^w13^ No clinical examination of individuals was conducted, thus, the presence of a lesion (ulcer) relied on girls’ reporting an itchy/sore vagina. We cannot rule out that girls when faced with intimate questions about vaginal discharge or pain may have been too embarrassed, or too scared, to disclose symptoms face-to-face in the nurses’ interview.^23^ They may also be less familiar with their bodily ‘norm’ so symptoms may have gone unnoticed. Moreover, due to financial constraints, examination of other RTI such as syphilis and HSV-2 was not possible and we recommend that studies include these also to ensure comprehensive mapping of infection, and healthcare needs of adolescent girls.

Synonymous with our findings, a study conducted in South Africa reported symptomatic vaginal discharge among high-risk girls and women was a poor predictor of RTI and genital tract inflammation (sensitivity of 12.3% and specificity of 93.8%), with 87.7% of laboratory-diagnosed infections having no accompanying clinical symptoms.^24^ Similarly, a study in Botswana reported three-quarters of pregnant women attending antenatal clinics were asymptomatic.^25^ Syndromic management failed to capture half of women with *T. vaginalis* or bacterial vaginosis infections during antenatal care, leading to significant challenges for diagnosis and treatment among this critically vulnerable group.^25^ Adding to the evidence from South Africa^26^ and Botswana,^25^ ^27^ our data suggest that the syndromic approach is not a good enough indicator of RTI, and new approaches are needed.

A study among African females found a high prevalence of bacterial vaginosis was associated with recent unprotected sexual intercourse.^26^ Among adolescent girls from South Africa and Kenya, 16–17-year-old sexually active girls reported minimal symptoms (2/60; 3.3%) while the prevalence of laboratory-confirmed RTI was very high (in 60 girls, 23 were diagnosed with bacterial vaginosis, 12 with *C. albicans*, 4 with *T. vaginalis* and 4 with *C. trachomatis*).^26^ ^28^ ^w14^

The relationship between bacterial vaginosis, *N. gonorrhoeae*, *C. trachomatis* and genital ulcer disease, and the increase in the risk of HIV transmission,^3^ ^5–8^ ^w2–3^ makes early detection and diagnosis of RTI a major public health imperative.^2^ ^w15^ Studies in western Kenya among the Luo show a rapid rise in HIV and HSV-2 prevalence during adolescence among girls,^11^ suggesting that many of our schoolgirls not already infected with HIV are at further risk of acquiring HIV due to their RTI-infected status. A reduction in prevalence is possible, for example, in Tanzania in areas where women had improved access to laboratory diagnosis and treatment of RTIs, incident HIV infections were reduced by 40%.^29^ *T. vaginalis* infection is a common curable sexually transmitted infection with high prevalences observed in the African subcontinent.^2^ However, this vaginal condition may cause substantial morbidity among women in developing countries if untreated. No comparison study was available among adolescents; however, among pregnant women in Botswana, *T. vaginalis* was found in 19% and bacterial vaginosis in 38% of the study participants.^25^ Unless drastic measures are put into place, the high prevalence of infections found in young adolescent girls is likely to remain undetected and untreated, leading to a rising pool of infection in the local population, increasing morbidity and reducing their fertility, as well as increasing their risk of other RTI and HIV.

From our data, we have identified three priority areas for public health intervention among this female adolescent population. First, girls need to learn self-awareness of their own bodies to be able to recognise any changes in order that symptoms are detected as being different from their normal state. Second, girls need to understand that it is possible to have an RTI despite having no symptoms. Third, methods to achieve prevention must be made readily available to adolescent girls. Our data, combined with other findings, point to the urgent need for sexual and reproductive health education, including a focus on personal hygiene, combined with the provision of friendly sexual and reproductive health services including treatment and counselling services. The latter must be easily accessible to girls who have little free time and no resources to travel**.** Without such interventions, it is difficult to envisage any real progress in reducing the prevalence of RTI, poor maternal health outcomes and HIV acquisition.^3^ ^w15^ One solution, in part, may be school-based,^30^ through school nurses who, as well as treating and referring girls to specialist services, could educate and counsel girls as they reach menarche and become sexually active.

In summary, our study adds to a sparse body of literature, showing a high prevalence of RTI among school-aged girls in a rural African setting. The majority of infections were asymptomatic, demonstrating a very poor sensitivity of symptom-based reporting. The use of syndromic management to reduce RTI and HIV incidence in this adolescent population would leave most of these very vulnerable girls untreated (even if they would present to a health clinic). Technological advancements that can enable quick, easy and cost-effective diagnosis of RTIs may be a desirable step towards realising effective management of reproductive health in rural African settings.

**Key messages**

- Adolescent schoolgirls in rural Kenya have a high prevalence of reproductive tract infections.
- Symptom-based reporting has a poor sensitivity missing three-quarters of laboratory-confirmed infections.
- Greater public health efforts are required to diagnose and treat infections among vulnerable girls to minimise long-term sequelae.

## Supplementary Material

Web supplement 1

Web supplement 2

Web supplement 3

## References

[1] WHO. Report on global sexually transmitted infection surveillance. Geneva, Switzerland: World Health Organization, 2013.

[2] WHO. Global prevalence and incidence of selected curable sexually transmitted infections. Geneva, Switzerland: World Health Organization, 2001.

[3] Cohen CR, Lingappa JR, Baeten JM, et al Bacterial vaginosis associated with increased risk of female-to-male HIV-1 transmission: a prospective cohort analysis among African couples. PLoS Med 2012;9:e1001251 10.1371/journal.pmed.1001251 22745608 PMC3383741

[4] Mitchell C, Balkus JE, Fredricks D, et al Interaction between lactobacilli, bacterial vaginosis-associated bacteria, and HIV Type 1 RNA and DNA Genital shedding in U.S. and Kenyan women. AIDS Res Hum Retroviruses 2013;29:13–19. 10.1089/AID.2012.0187 23020644 PMC3537306

[5] Balkus JE, Richardson BA, Rabe LK, et al Bacterial vaginosis and the risk of trichomonas vaginalis acquisition among HIV-1-negative women. Sex Transm Dis 2014;41:123–8. 10.1097/OLQ.0000000000000075 24413493 PMC4128240

[6] Cherpes TL, Meyn LA, Krohn MA, et al Association between acquisition of herpes simplex virus type 2 in women and bacterial vaginosis. Clin Infect Dis 2003;37:319–25. 10.1086/375819 12884154

[7] Wiesenfeld HC, Hillier SL, Krohn MA, et al Bacterial vaginosis is a strong predictor of Neisseria gonorrhoeae and Chlamydia trachomatis infection. Clin Infect Dis 2003;36:663–8. 10.1086/367658 12594649

[8] Gillet E, Meys JF, Verstraelen H, et al Bacterial vaginosis is associated with uterine cervical human papillomavirus infection: a meta-analysis. BMC Infect Dis 2011;11:10 10.1186/1471-2334-11-10 21223574 PMC3023697

[9] Caixeta RC, Ribeiro AA, Segatti KD, et al Association between the human papillomavirus, bacterial vaginosis and cervicitis and the detection of abnormalities in cervical smears from teenage girls and young women. Diagn Cytopathol 2015;43:780–5. 10.1002/dc.23301 26173042

[10] Yi TJ, Shannon B, Prodger J, et al Genital immunology and HIV susceptibility in young women. Am J Reprod Immunol 2013;69(Suppl 1):74–9. 10.1111/aji.12035 23157424

[11] Amornkul PN, Vandenhoudt H, Nasokho P, et al HIV prevalence and associated risk factors among individuals aged 13–34 years in Rural Western Kenya. PLoS ONE 2009;4:e6470 10.1371/journal.pone.0006470 19649242 PMC2714463

[12] Buve A, Weiss HA, Laga M, et al The epidemiology of trichomoniasis in women in four African cities. AIDS 2001;15(Suppl 4):S89–96. 10.1097/00002030-200108004-00010 11686470

[13] Buve A, Weiss HA, Laga M, et al The epidemiology of gonorrhoea, chlamydial infection and syphilis in four African cities. AIDS 2001;15(Suppl 4):S79–88. 10.1097/00002030-200108004-00009 11686469

[14] WHO. Guidelines for the management of sexually transmitted infections. Geneva, Switzerland: World Health Organisation, 2003.

[15] Romoren M, Hussein F, Steen TW, et al Costs and health consequences of chlamydia management strategies among pregnant women in sub-Saharan Africa. Sex Transm Infect 2007;83:558–66. 10.1136/sti.2007.026930 17932126 PMC2598644

[16] Das P, Baker KK, Dutta A, et al Menstrual Hygiene Practices, WASH Access and the Risk of Urogenital Infection in Women from Odisha, India. PLoS ONE 2015;10:e0130777 10.1371/journal.pone.0130777 26125184 PMC4488331

[17] Alexander K, Oduor C, Nyothach E, et al Water, sanitation and hygiene conditions in Kenyan rural schools: are schools meeting the needs of menstruating girls? Water 2014;6:1453–66. 10.3390/w6051453

[18] Tanksale VS, Sahasrabhojanee M, Patel V, et al The reliability of a structured examination protocol and self administered vaginal swabs: a pilot study of gynaecological outpatients in Goa, India. Sex Transm Infect 2003;79:251–3. 10.1136/sti.79.3.251 12794216 PMC1744681

[19] Hallfors D, Cho H, Rusakaniko S, et al Supporting adolescent orphan girls to stay in school as HIV risk prevention: evidence from a randomized controlled trial in Zimbabwe. Am J Public Health 2011;101:1082–8. 10.2105/AJPH.2010.300042 21493943 PMC3093274

[20] Hargreaves J, Morison L, Kim J, et al The association between school attendance, HIV infection and sexual behaviour among young people in rural South Africa. J Epidemiol Community Health 2008;62:113–19. 10.1136/jech.2006.053827 18192598

[21] Phillips-Howard P, Olilo G, Burmen B, et al Menstrual needs and associations with sexual and reproductive risks in rural Kenyan females: a cross-sectional behavioural survey linked with HIV prevalence. J Womens Health 2015;24:1–11. 10.1089/jwh.2014.5031 PMC4624246 26296186

[22] Hewett PC, Mensch BS, Erulkar AS Consistency in the reporting of sexual behaviour by adolescent girls in Kenya: a comparison of interviewing methods. Sex Transm Infect 2004;80(Suppl 2):ii43–8. 10.1136/sti.2004.013250 15572639 PMC1765856

[23] Plummer ML, Wight D, Ross DA, et al Asking semi-literate adolescents about sexual behaviour: the validity of assisted self-completion questionnaire (ASCQ) data in rural Tanzania. Trop Med Int Health 2004;9:737–54. 10.1111/j.1365-3156.2004.01254.x 15189466

[24] Mlisana K, Naicker N, Werner L, et al Symptomatic vaginal discharge is a poor predictor of sexually transmitted infections and genital tract inflammation in high-risk women in South Africa. J Infect Dis 2012;206:6–14. 10.1093/infdis/jis298 22517910 PMC3490689

[25] Romoren M, Velauthapillai M, Rahman M, et al Trichomoniasis and bacterial vaginosis in pregnancy: inadequately managed with the syndromic approach. Bull World Health Organ 2007;85:297–304. 10.2471/BLT.06.031922 17546311 PMC2636319

[26] Jespers V, Crucitti T, Menten J, et al Prevalence and correlates of bacterial vaginosis in different sub-populations of women in sub-Saharan Africa: a cross-sectional study. PLoS ONE 2014;9:e109670 10.1371/journal.pone.0109670 25289640 PMC4188821

[27] Romoren M, Sundby J, Velauthapillai M, et al Chlamydia and gonorrhoea in pregnant Batswana women: time to discard the syndromic approach?. BMC Infect Dis 2007;7:27 10.1186/1471-2334-7-27 17437632 PMC1955709

[28] Jespers V, van de Wijgert J, Cools P, et al The significance of Lactobacillus crispatus and L. vaginalis for vaginal health and the negative effect of recent sex: a cross-sectional descriptive study across groups of African women. BMC Infect Dis 2015;15:115 10.1186/s12879-015-0825-z 25879811 PMC4351943

[29] Grosskurth H, Mosha F, Todd J, et al Impact of improved treatment of sexually transmitted diseases on HIV infection in rural Tanzania: randomised controlled trial. Lancet 1995;346:530–6. 10.1016/S0140-6736(95)91380-7 7658778

[30] Plummer ML, Wight D, Wamoyi J, et al Are schools a good setting for adolescent sexual health promotion in rural Africa? A qualitative assessment from Tanzania. Health Educ Res 2007;22:483–99. 10.1093/her/cyl099 17018766

