# Supplementary material for: Prevalence of reproductive tract infections and the predictive value of girls’ symptom-based reporting: findings from a cross-sectional survey in rural western Kenya
Source: Sex Transm Infect. 2016 Jan 27;92(4):251–6. doi: 10.1136/sextrans-2015-052371 (PMC4893088; doi:10.1136/sextrans-2015-052371)
Supplement: Web supplement 1 [file sextrans-2015-052371-s1.pdf]

4247247493

MS-STUDY
RTI SCREENING FORM

File number [| | | |]

○ Gem

Girl's study ID [|] - [ ] - [|]   Nurse ID [|]   Visit date [|] / [|] / [| | |]

SECTION 1:CLINICAL CHECK-(SCREENING FOR ILLNESS)

1.**Be isebedo kiwinjo maber/kingima e dwe mokalo ni?** (Have you been feeling well this last month?) *feel_well*
○ Ee (Yes) ○ Ooyo (No)

2.**Ka ooyo,nitimang'o?**(If no,what did you do) *feel_other*
○ Dhi ei osiptal(Go to hospital) ○ Dhi ei od thieth(Go to health facility) ○ bara skul(Miss school) ○ Mamoko(Other)

3.If any health problems that are not menstrual/RTI/TSS,write in this box what the problem was,how you advised care and what services are signposted: *health_problems*

____

____

4. **Be isebedo kichuero remo mang'eny e kinde manidhiye e dwe mogik?**(Have you been bleeding very heavily during your recent monthly period?) *bleed_heavily*
○ Ee (Yes) ○ Ooyo (No)

5. **Be isebetie kineno remo bang dwe mogik kapok ichako dwe manyien?**(Have you had any bleeding in between one period and the next period?) *bleed_btwn*
○ Ee (Yes) ○ Ooyo (No)

6. **Saa asaya e dwe mokalo-Bende iwinjo rem kata lit sama iolo pii/layo?**(Do you have pain or burning during urination?) *burn_urine*
○ Ee (Yes) ○ Ooyo (No)

7. **Saa asaya e dwe mokalo-Bende isebedo kilayo sate sate/saa ka saa(layo mohingo kaka pile)?**(Do you have more frequent urination(pass urine more than usual)? *freq_urine*
○ Ee (Yes) ○ Ooyo (No)

8. **Saa asaya e dwe mokalo-Be iwinjoga rem e piny iyi,kata e duong'ni)?**(Do you have any pain in your abdomen,or vagina?) *abdomen_pain*
○ Ee (Yes) ○ Ooyo (No)

9. **Saa asaya e dwe mokalo-Be duong'ni ili?** (Any time last month-Are you at all itchy,sore in your vagina?) *itchy_private*
○ Ee (Yes) ○ Ooyo (No)

10. **Saa asaya e dwe mokalo-Be nitie tik moro amora mawuok e duong'ni?**(Is there any smell coming from your vagina?) (If No go to Q12) *smell_coming*
○ Ee (Yes) ○ Ooyo (No)

11. **ka kamano ,ler ni en tik machal nadi**(If yes,describe type of smell) *smell_other*
○ Dirty ○ Fishy ○ Blood ○ Other

12. **Saa asaya e dwe-Be nitiega gik mawuok/machuer kowuok e duog'ni?**(Is there any discharge coming from your vagina?) (If No go to Q15) *disch_coming*
○ Ee (Yes) ○ Ooyo (No)

13. **ka kamano ,en**(If yes,is it:) *is_it*
○ Machal go othinyo(Mucusy) ○ Motimo byoyo(Frothy) ○ Mong'inore(curd-like) ○ NA ○ Other

14. **ka kamano ,no chal nadi**(If yes,what color:) *what_color*
○ Rachar(White) ○ Majan(Green) ○ Raton'g(Yellow) ○ Motimo remo(Bloody) ○ NA

15. **Bende ne iriwrie gi ng'ama dichuo e dwe mokalo?**(Did you have sex in the past month?) (If No go to Q18) *have_sex*
○ Ee (Yes) ○ Ooyo (No)

16. **Ka kamano,be nenitiere chuer,rem kata chandruok moro amora kaluwore gi riwruok?** If yes,was there any bleeding,pain or other problem with sex?) *pain_sex*
○ Ee (Yes) ○ Ooyo (No) ○ NA

17. **Ka kamano,ne en ang'o?**(If yes,What?) *yes_what*
○ Ee,rem (Yes),pain ○ Ee ,chuer(Yes,bleed) ○ Mamoko,(Other) ○ NA

ACTIONS

18.**SWAB TAKEN:** *swab_taken* ○ Yes ○ No

Time collected (24HR)

TIME: [|] - [|] *time_swab*

Verified [ ]
