# Supplementary material for: Prevalence of reproductive tract infections and the predictive value of girls’ symptom-based reporting: findings from a cross-sectional survey in rural western Kenya
Source: Sex Transm Infect. 2016 Jan 27;92(4):251–6. doi: 10.1136/sextrans-2015-052371 (PMC4893088; doi:10.1136/sextrans-2015-052371)
Supplement: Web supplement 2 [file sextrans-2015-052371-s2.pdf]

Table S1 Number and percentage of overlapping symptoms reported by girls

	Heavy menstruation	Between menses bleeding	Burning urine	Frequency urine	Abdominal/ vaginal pain	Pain/bleed during intercourse	Itchy/sore vagina	Malodorous smell	Vaginal discharge
Heavy menstruation (33) and	33 (100)	8 (24.2)	3 (9.1)	4 (12.1)	11 (33.3)	1 (3.0)	6 (18.2)	3 (9.1)	8 (24.2)
Between menses bleeding (20) and	8 (40.0)	20 (100)	2 (10.0)	3 (15.0)	5 (25.0)	0	2 (10.0)	1 (5.0)	6 (30.0)
Burning urine (11) and	3 (27.3)	2 (18.2)	11 (100)	3 (27.3)	7 (63.6)	1 (9.1)	6 (54.5)	2 (18.2)	1 (9.1)
Frequency of urine (13) and	4 (30.8)	3 (23.1)	3 (23.1)	13 (100)	3 (23.1)	0	5 (38.5)	4 (30.8)	2 (15.4)
Abdominal / vaginal pain (45) and	11 (24.4)	5 (11.1)	7 (15.6)	3 (6.7)	45 (100)	1 (2.2)	9 (20.0)	3 (6.7)	12 (26.7)
Pain/bleed during intercourse (2) and	1 (50.0)	0	0	0	1 (50.0)	2 (100)	1 (50.0)	1 (50.0)	1 (50.0)
Itchy/sore vagina (22) and	6 (27.3)	2 (9.1)	6 (27.3)	5 (22.7)	9 (40.9)	1 (4.5)	22 (100)	7 (31.8)	9 (40.9)
Malodorous smell (9) and	3 (33.3)	1 (11.1)	2 (22.2)	4 (44.4)	3 (33.3)	1 (11.1)	7 (77.7)	9 (100)	6 (66.6)
Vaginal discharge (53) and	8 (15.1)	6 (11.3)	1 (1.9)	2 (3.8)	12 (22.6)	1 (1.9)	9 (17.0)	6 (11.3)	53 (100)
