# Supplementary material for: Prevalence of reproductive tract infections and the predictive value of girls’ symptom-based reporting: findings from a cross-sectional survey in rural western Kenya
Source: Sex Transm Infect. 2016 Jan 27;92(4):251–6. doi: 10.1136/sextrans-2015-052371 (PMC4893088; doi:10.1136/sextrans-2015-052371)
Supplement: Web supplement 3 [file sextrans-2015-052371-s3.pdf]

S3 Additional Web References

w1 Sewankambo N, Gray RH, Wawer MJ, et al. HIV-1 infection associated with abnormal vaginal flora morphology and bacterial vaginosis. Lancet 1997;**350**(9077):546-50.

w2 Atashili J, Poole C, Ndumbe PM, et al. Bacterial vaginosis and HIV acquisition: a meta-analysis of published studies. AIDS 2008;**22**(12):1493-501.

w3 Madhivanan P, Krupp K, Chandrasekaran V, et al. Prevalence and correlates of bacterial vaginosis among young women of reproductive age in Mysore, India. Indian journal of medical microbiology 2008;**26**(2):132-7.

w4 WHO. The sexual and reproductive health of younger adolescents: research issues in developing countries. Geneva, Switzerland: World Health Organization, 2011.

w5 KAIS. Kenya AIDS Indicator Survey 2012. Preliminary Report ed. Nairobi, Kenya: National AIDS and STI Control Programme, Ministry of Health, 2013.

w6 WHO. Sexually transmitted infections and other reproductive tract infections- a guide to essential practice. Geneva, Switzerland World Health Organisation, 2005.

w7 Otieno FO, Ndivo R, Oswago S, et al. Correlates of prevalent sexually transmitted infections among participants screened for an HIV incidence cohort study in Kisumu, Kenya. Int J STD AIDS 2015;**26**(4):225-37.

w8 Garg R, Goyal S, Gupta S. India moves towards menstrual hygiene: subsidized sanitary napkins for rural adolescent girls - issues and challenges. Maternal and Child Health Journal 2012;**16**:767-74.

w9 Odhiambo FO, Laserson KF, Sewe M, et al. Profile: The KEMRI/CDC Health and Demographic Surveillance System--Western Kenya. Int J Epidemiol 2012;**41**(4):977-87.

w10 CDC. Diseases and related conditions: Sexually transmitted diseases US Centers for Disease Control and Prevention 2012 http://www.cdc.gov/std/general/default.htm (last accessed 28 November 2015).

w11 Rogstad K. *ABC of Sexually Transmitted Infections*. 6th ed: BMJ Books, 2011.

w12 Strauss RA, Eucker B, Savitz DA, et al. Diagnosis of bacterial vaginosis from self-obtained vaginal swabs. Infect Dis Obstet Gynecol 2005;**13**(1):31-5.

w13 Plummer ML, Ross DA, Wight D, et al. "A bit more truthful": the validity of adolescent sexual behaviour data collected in rural northern Tanzania using five methods. Sex Transm Infect 2004;**80 Suppl 2**:ii49-56.

w13 Kyongo JK, Crucitti T, Menten J, et al. Cross-Sectional Analysis of Selected Genital Tract Immunological Markers and Molecular Vaginal Microbiota in Sub-Saharan African Women, with Relevance to HIV Risk and Prevention. Clin Vaccine Immunol 2015;**22**(5):526-38.

w14 Buve A, Jespers V, Crucitti T, et al. The vaginal microbiota and susceptibility to HIV. AIDS 2014;**28**(16):2333-44.
